# Model for the Correlation between Anodic Dissolution Resistance and Crystallographic Texture in Pipeline Steels

**DOI:** 10.3390/ma11081432

**Published:** 2018-08-14

**Authors:** Manuel Madrigal-Cano, Luis Hernández-Maya, José Manuel Hallen, Mónica Corrales-Luna, Elsa Miriam Arce-Estrada, Tu Le Manh

**Affiliations:** Instituto Politécnico Nacional, Departamento de Ingeniería en Metalurgia y Materiales, UPALM Edificio 7, CDMX 07738, México; mrmcano@hotmail.com (M.M.-C.); luisgerardo.hm2016@gmail.com (L.H.-M.); j_hallen@yahoo.com (J.M.H.); monis_c5@hotmail.com (M.C.-L.); earce@ipn.mx (E.M.A.-E.)

**Keywords:** corrosion resistance index, anodic dissolution, crystallographic texture, pipeline steels, material selection

## Abstract

This paper presents a novel physical–mathematical model to describe the relationship between the crystallographic texture and corrosion behavior of American petroleum institute (API) 5L steels. Symmetric spherical harmonic functions were used to estimate the material’s corrosion resistance from crystallographic texture measurements. The predictions of the average corrosion resistance index made from the crystallographic texture were in good agreement with those obtained from potentiodynamic polarization and electrochemical impedance spectroscopy measurements for the studied steels. This agreement validates the capacity of this model and opens the possibility of applying it as a novel criterion for materials selection and design stages to combat corrosion problems.

## 1. Introduction

Although the corrosion problems of steels have been extensively investigated in the literature [1,2], modelling the relationship between crystallographic texture and corrosion is not straightforward. It is known that the correct selection of materials can be an important starting point and tool for reducing the corrosion effect during the stages of structure construction and design. A common way to evaluate the corrosion activity of metals is through the corrosion potential and current density (*E*_corr_ and *j*_corr_), and the anodic and cathodic Tafel slopes (*b_a_* and *b_c_*), obtained by polarization techniques [3,4,5]. However, these experimental techniques have presented issues, including blindness, workload concerns, time and resource waste [6], and variations in the results, the latter of which is due to strong dependence on sample surface state and other metallurgical variables of the material, namely chemical composition and microstructure [7,8,9]. In order to solve this problem, it is necessary to evaluate and quantify the corrosion resistance of the material by other means.

It has been experimentally verified [9,10,11,12,13,14,15,16,17,18] that some metallurgical parameters, such as grain size, grain boundary, and their distributions, as well as surface roughness, crystallographic planes, and grain orientations, have a strong influence on the anodic dissolution of the metal. This, in fact, refers to the anisotropy of corrosion, which in a crystal can be defined as the dependence of the dissolution velocity of metals on the crystallographic directions [18]. Meanwhile, in a polycrystal, this behavior depends on the crystallographic texture. This is because texture develops in alloys and metals during their mechanical deformation, such as in rolling, forging, and drawing, and the established preferred orientations can introduce significant change to the material properties [19]. The considerable effect of the crystallographic texture on the corrosion process has been studied in several works [20,21,22]. Venegas et al. explained that the formation of the texture components {111}|| ND (Normal direction) fibers, developed by warm rolling, could reduce the susceptibility to hydrogen induced crackinging (HIC), while hot-rolled specimens with nearly random texture or the strong {001}||ND texture demonstrated less resistance to HIC [22]. In spite of this fact, a generalized model capable of describing the link between the crystallographic texture and corrosion behavior of metals (anodic dissolution) has been lacking.

Shahryari et al. [23] proposed a correlation between the pitting corrosion resistance and the crystallographic orientation, wherein the susceptibility of the surface of SS316LVM stainless steel to pitting corrosion (the pitting susceptibility index) was determined from electron back-scatter diffraction (EBSD) data by way of generalized spherical harmonics. Nevertheless, this model had several limitations, namely that it did not include the sample symmetry, it only used steel samples with one type of texture, the number of coefficients of the harmonic function was small, and the information obtained from EBSD was not statistically significant (in comparison with the X-ray macro-texture technique) to completely describe the dependence of crystallographic orientations on the anodic dissolution of the material. Meanwhile, in another approach, Venegas et al. [24] studied the influence of crystallographic texture, measured by X-ray diffraction, on pitting corrosion in low carbon steels. They suggested that the ratio of the volume fraction of the beneficial (high-resistance) texture fibers to the volume fraction of the detrimental (low-resistance) fibers could be a good measure for the pitting susceptibility of the material. However, this model could not establish a direct relationship between the pitting corrosion and the crystallographic texture, since the beneficial/detrimental texture components and their volume fraction must be known previously and carefully analyzed. In this work, the main interest lies in the anisotropy of the anodic dissolution resistance of polycrystalline materials. Moreover, a property of a polycrystal must be properly determined by averaging that over the orientation distribution function (ODF) [25], rather than using the volume fraction.

Therefore, in this work, a new model is proposed, based on the concept provided by Sharyaria et al. [23], for the correlation between the anodic dissolution resistance and the crystallographic texture of three steels, of American petroleum institute (API) 5L specifications, with different microstructures and textures. This proposed model allowed us to predict the average corrosion resistance index (CRI) of API 5L steels from the crystallographic texture information using symmetric spherical surface harmonics. The average CRI predictions made from the X-ray texture measurements were validated with the experimental results obtained from potentiodynamic polarization and electrochemical impedance spectroscopy (EIS) techniques.

## 2. Materials and Methods

### 2.1. Materials

Three API 5L steel samples (X56, X52, and X60), extracted from out-of-service pipelines, were used to explore the relationship between the crystallographic texture and the corrosion behavior. Three samples of each steel type were obtained in the form of disks with dimensions of 1 cm in diameter and 2.5 mm in thickness on the rolling plane (RD–TD, Hereafter the notations RD, TD, and ND refer to the rolling, transverse, and normal directions of the pipe) and transverse planes (TD–ND and RD–ND). The chemical composition of the studied steels can be seen elsewhere in reference [25]. All of the aforementioned disk-shaped samples were mechanically prepared using SiC grit papers, polished to 0.1 µm using diamond spray, then polished with colloidal silica down to 20 nm. This latter step was carefully carried out to avoid any influence of the under-the-surface deformation—introduced by the grinding/polishing steps—on the experimental measurements. A microstructure study was also carried out using a scanning electron microscope (SEM JEOL JSL-6300, Tokyo, Japan).

### 2.2. Crystallographic Texture Measurements

The texture measurements of the steel samples were performed with an X-ray diffractometer (Bruker D8 Advance, Karlsruhe, Germany) with Cu Kα radiation, coupled with an Euler cradle. Three incomplete pole figures {011}, {002}, and {112} were measured on the rolling plane for each steel sample. These experimental pole figures were analyzed using the arbitrary defined cell (ADC) method [26], implemented in LaboTex software (Version 3.0, LaboSoft, Krakow, Poland), to calculate the orientation distribution function (ODF). From this ODF, the inverse pole figure (IPF) of the ND was determined for each steel sample.

### 2.3. Electrochemical Tests

The potentiodynamic polarization and EIS measurements were performed at room temperature in an EG&G-PAR potentiostat/galvanostat Model 263A (Princeton Applied Research, Oak Ridge, TN, USA), using a water-jacketed cell composed of three electrodes. An API 5L steel disk-shaped sample embedded in epoxy resin, with an area of 0.7854 cm^2^, was used as the working electrode (WE); a graphite bar and a saturated calomel electrode (SCE) were used as the counter and reference electrode, respectively. These experiments were performed in triplicate on the same steel samples (surfaces), which were previously measured by the X-ray texture diffraction. The solution, which was selected, according to Espina-Hernández et al. [27], to simulate the corrosive environment of pipelines, was prepared using deionized water with the appropriate amount of NaCl and NaSO_4_ to obtain the desired composition. The main ion concentrations of this solution are shown in Table 1.

The potentiodynamic polarization curves were recorded from −250 to +250 mV in relation to the open circuit potential (OCP), at a scan rate of 0.16 mV s^−1^ after 30 min of immersion, which was the time necessary for the OCP to remain constant for all of the steel samples. 

EIS measurements were carried out at room temperature using frequencies between 100 mHz to 100 kHz, with an alternating current (AC) amplitude of 10 mV peak to peak from OCP. All of the potentials which are reported in this work are with respect to the SCE.

## 3. Model for Estimating the Average Corrosion Resistance Index from Crystallographic Texture

It is known that corrosion is an interfacial phenomenon [28]. For this reason, understanding the surface state and its associated properties (surface roughness, planar density) can be a good indicator of the corrosion susceptibility of a family plane {*hkl*} parallel to that metal surface, since crystalline defects can be active sites for corrosion, nucleation and growth mechanisms.

Venegas et al. [24] showed that the resistance to the dissolution anodic (*R*_{*hkl*}_), as a function of point density (*ρ*_{*hkl*}_) associated with crystallographic planes {*hkl*} exposed to the surface of the metal, could be determined by:

(1) $$R_{\left\{hkl\right\}}=\frac{\rho_{\left\{hkl\right\}}}{\rho_{\left\{110\right\}}}=\frac{\sqrt{2}}{\sqrt{h^{2}+k^{2}+l^{2}}}$$

Subjected to the constraint: *h* + *k* + *l* = 2*n*.

Where *ρ*_{110}_ is the point density for the plane {110} for body centered cubic (BCC) structure and *ρ*_{*hkl*}_ is the point density for the plane {*hkl*}, defined by:

(2) $$\rho_{\left\{hkl\right\}}=\frac{2}{a_{0}{}^{2}\sqrt{h^{2}+k^{2}+l^{2}}};\;h+k+l=n$$

where *a*_0_, the lattice parameter, was equal to 2.866 Å for pure BCC iron. All of them, hereafter, must satisfy the constraint *h* + *k* + *l* = 2*n* ∀ *n* ∈ *N* for this kind of lattice.

Additionally, Blonski et al. [29] found that the relationship between surface roughness (*S_r_*) and crystallographic orientation can be given by:

(3) $$S_{r}=\frac{A_{2D}}{A_{Fe}}=\frac{8}{3\pi }\sqrt{h^{2}+k^{2}+l^{2}}$$

(4) $$A_{2D}=\frac{a_{0}{}^{2}}{2}\sqrt{h^{2}+k^{2}+l^{2}}$$

(5) $$A_{Fe}=\frac{3}{16}\pi a_{0}^{2}$$

where *A_2D_* is the area of the surface unit cell and *A_Fe_* is the cross-sectional area of a Fe atom in a BCC lattice.

From Equations (2) and (3), $\rho_{\left\{hkl\right\}}$ can be related to *S_r_* by the following expression:

(6) $$\;\rho_{\left\{hkl\right\}}\propto \frac{1}{S_{r}}$$

Therefore, from Equations (1) and (6), the relationship between *R*_{*hkl*}_ and *S_r_* can be established by the following proportionality:

(7) $$R_{\left\{hkl\right\}}\propto \frac{1}{S_{r}}$$

Equation (7) means that if the crystallographic plane, associated with the grain parallel to the surface, has less surface roughness, its resistance to the anodic dissolution is higher. Interestingly, this has been verified by Shahryari et al. [23] using the generalized spherical surface harmonics, which assists in the evaluation of the anisotropic nature of the corrosion behavior of stainless steel. Nevertheless, their approach did not consider the crystal–sample symmetry relationship. Therefore, it is more convenient to apply the function developed by Bunge, in which the symmetric spherical surface harmonics with cubic symmetry is given by [30]:

(8) $$E(h)=\sum_{l=4}^{l_{\max}}\sum_{\mu =1}^{M(l)}e_{l}^{\mu }{\overset{:}{k}}_{l}^{\mu }(h),$$

where $e_{l}^{\mu }$ is a series of constants for various values of *l*, which were calculated using the least-squared-fitting method [23] in Mathematica software.

The symmetric spherical harmonic function with cubic symmetry, ${\overset{:}{k}}_{l}^{\mu }(h)$, in Equation (8) can be determined by [30]:

(9) $${\overset{:}{k}}_{l}^{\mu }(h)={\overset{:}{k}}_{l}^{\mu }(\phi ,\beta )=\sum_{m=0}^{l}{\overset{:}{B}}_{l}^{m\mu }{\bar{P}}_{l}^{m}(\phi )\cos m\beta$$

where ${\overset{:}{B}}_{l}^{m\mu }$ is the symmetry coefficients for cubic symmetry; ${\bar{P}}_{l}^{m}\left(\phi \right)$ is the normalized, associated Legendre functions with ϕ (0°≤ϕ≤45°) and β (0°≤β≤53°) in the asymmetric triangle of the cubic crystalline system, and ***h*** = {*h*, *k*, *l*} is the direction normal to the crystallographic plane of interest with Miller indices {*hkl*}.

**Figure 1:**
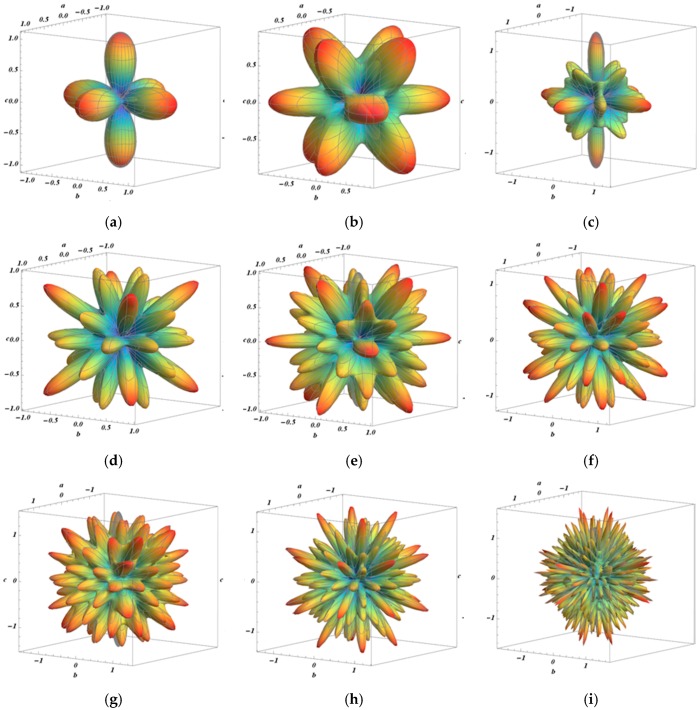
Rate of change of the symmetric surface harmonics function, of the order l, using Equation (9): (**a**) $k_{4}^{:1}$; (**b**) $k_{6}^{:1}$; (**c**) $k_{8}^{:1}$; (**d**) $k_{10}^{:1}$; (**e**) $k_{12}^{:2}$; (**f**) $k_{14}^{:1}$; (**g**) $k_{16}^{:2}$; (**h**) $k_{18}^{:2}$; and (**i**) $k_{34}^{:1}$.

The values of *l* in Equation (8) were chosen to extend to 22, instead of 10 as proposed by Shahryari et al. [23], due to the fact that a smaller *l* order—up to 10—would result in the rate of change of the harmonic function being very fast (Figure 1a–d), while with *l* > 10 the behavior of the rate of change would be closer to a real system (Figure 1e–i).

Taking into account Equations (7) and (8), the relationship between the anodic dissolution resistance, *E*(*h*), and surface roughness is:

(10) $$E\left(h\right)\propto \frac{1}{S_{r}},$$

It is important to stress that indeed Equation (10) gives physical meaning to the symmetric spherical surface harmonic function and the possibility to describe the anisotropy of corrosion in the function of any crystallographic planes. Henceforth, from Equation (10), *E*(*h*), which is called CRI, can be defined as a new anisotropy function of the anodic dissolution rate of the crystallographic planes parallel to the surface, that can be estimated over the entire angular range of the asymmetric triangle of the cubic crystal system (standard triangle due to the cubic symmetry).

**Figure 2:**
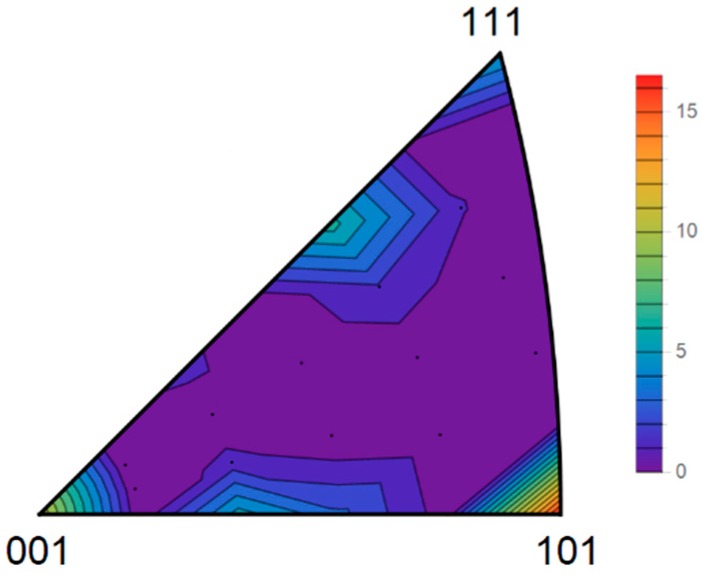
Anisotropy surface of the anodic dissolution of BCC materials calculated for any crystallographic plane.

For a better understanding of the benefits of Equation (10), the variation of the anodic dissolution resistance in the function of the crystallographic planes, described by *E*(*h*), is represented through the inverse pole figure of the cubic crystal system, as depicted in Figure 2. The results indicate that the tendency to suffer corrosion follow the sequence: {213} > {111} > {103} > {112} > {001} > {101}, which is in agreement with the trends observed in several works [7,8,10,11,14,15,19].

In order to study the corrosion property in a polycrystal, it is important to know the crystallographic texture or the distribution of crystal orientations in it. The average CRI, $\bar{E}\left(h\right)$, in a polycrystalline material with orientation distribution function (ODF), *f*(*g*), can be determined for any anodic dissolution vector (*Φ*, *β*) in the sample´s reference system [30]:

(11) $$\bar{E}\left(h\right)=\bar{E}\left(\Phi ,\beta \right)=\int_{ES}E\left(\Phi ,\beta ,g\right)f\left(g\right)dg,$$

where *g* is the crystal orientation and ES is the Euler space; *f*(*g*) should be determined considering the cubic (crystal)–orthorhombic (sample) symmetry.

Since corrosion starts on the metal surface rather than the entire volume of the material [28], the average CRI of interest in a polycrystal is in the direction normal to the rolling plane of the sample. Under this condition, $\bar{E}\left(h\right)$ in a polycrystal can be estimated by [23,30]:

(12) $$\bar{E}\left(h\right)\propto \oint^{\text{​}}\int^{\text{​}}E\left(\Phi ,\beta \right)IPF\left(\Phi ,\beta \right)\sin\left(\Phi \right)d\Phi d\beta ,$$

where *IPF*(*Φ*, *β*) is the intensity of the inverse pole figure from the X-ray diffraction measurements.

Equation (12) proposes a new model for estimating the average CRI in API 5L steels from the crystallographic texture measurements. This will be evaluated in steel samples with different microstructures and textures for further comparison with the results obtained from the electrochemical tests.

## 4. Results and Discussion

### 4.1. Microstructure Study

**Figure 3:**
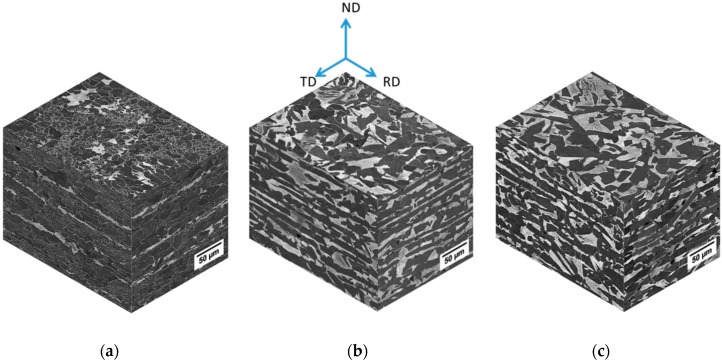
SEM micrographs of the studied steels: (**a**) X56, (**b**) X52, and (**c**) X60. The ferrite and pearlite phases are represented by the dark and bright colors, respectively.

Figure 3 shows the SEM micrographs represented in the form of a cubic crystal for the three studied steels (X56, X52, and X60). In general, these steels have a ferritic/pearlitic microstructure with different grain size. The equiaxial grain morphology was observed on the rolling plane with a relatively homogeneous distribution, while elongated grains were observed on the transverse (RD–ND and TD–ND) planes due to the pipe manufacturing process [31,32,33]. Microstructural parameters such as grain size and volume fraction of the ferrite phase were determined by optical microscopy, as shown in Table 2. The inclusion (MnS) content in these steels was insignificant according to the work published on pipeline steels [22].

### 4.2. Estimation of the Average CRI from Crystallographic Texture

**Figure 4:**
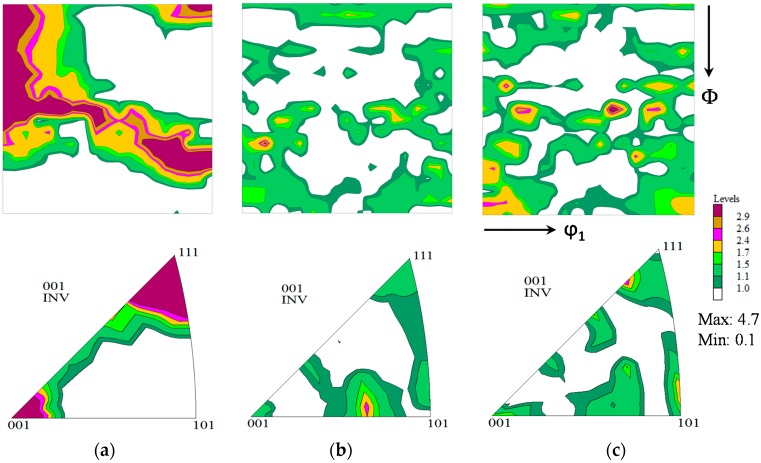
ϕ_2_ = 45° section of the orthorhombic orientation distribution functions (ODFs) and their corresponding inverse pole figure (IPF) of ND of the studied steel samples before polarization tests: (**a**) X56; (**b**) X52; (**c**) X60. The angles (Φ and ϕ_2_) ranged within 0°–90° in the Euler space.

Figure 4 shows the cubic–orthorhombic representation of the X-ray-derived ODF of the studied steels in the ϕ_2_ = 45 section of Euler space before the electrochemical tests [30]. It can be seen that the crystallographic texture of these steels was characterized by the presence of the {100}||ND, {111}||ND, {112}||ND, and {110}||ND texture fibers with different degrees of development [24,32,33]. The volume fractions (*V*_{*hkl*}_ND) of these fibers were calculated as shown in Table 3. The formation of these texture fibers can be explained as the consequence of the hot rolling process during manufacture of the pipe [31,32,33]. X52 and X60 steels (Figure 4b,c) showed a texture close to random, with a relatively small degree of difference, while the X56 steel (Figure 4a) presented a markedly strong texture dominated by {112}||ND and {111}||ND fibers. These differences in texture could influence the electrochemical behavior of these steel samples and, consequently, their corrosion resistance.

As previously explained, due to the surface behavior of corrosion phenomena, it is more convenient to use data from the IPFs of ND. In fact, the representation of texture through the IPFs is a direct way to exhibit the distribution of the crystallographic planes of the grains exposed to the surface sample (crystallographic planes perpendicular to the steel sample surface). Data obtained from these IPFs were used to calculate the average CRI, $\bar{E}\left(h\right)$, which is summarized in Table 4. The results indicate that the corrosion resistance of the studied steels followed the order of: X56 < X52 < X60, since the higher the CRI values, the better the corrosion resistance. According to the previous texture analysis, this order is logical, as the high volume fraction of {112}||ND and {111}||ND fibers (see Table 3) in X56 steel clearly indicates that this material possesses a looser structure (low density planes) than the other steels (X52 and X60). X52 and X60 contain higher volume fractions of {110}||ND fibers, which generate the most compact (dense) planes in BCC materials. Therefore, X56 steel may dissolve at a higher rate than X52 and X60 steels. This effect can be clearly observed through the illustration of the anisotropy surface of the anodic dissolution, shown in Figure 2. It is important to note that this analysis only helps when the texture is markedly different—in terms of both the texture components and their magnitude—from one material to the next, such as in X56 and X60 steel, but it is notoriously difficult when the texture is similar, as can be seen in the case of the X52 and X60 steels. Nevertheless, the model proposed in this paper could satisfactorily solve this problem, since Equation (12) deals with all of the texture data obtained from the IPFs of the material. For the sake of validation, the average CRI predictions made from the crystallographic texture were compared with those obtained from potentiodynamic polarization and EIS studies.

### 4.3. Model Validation

#### 4.3.1. Potentiodynamic Polarization Tests

In order to verify the validity of the proposed model, parameters that represent the corrosion resistance of the three steels (X56, X52, and X60) were determined by the potentiodynamic polarization method, and compared to the predictions made from the crystallographic texture measurements. It is important to stress that, according to Fushimi et al. [10], the anisotropic corrosion of iron can be satisfactorily studied by means of the potentiodynamic polarization technique, as well as experimentally revealed by assessing the dependence of the crystallographic orientation on the corrosion resistance of the material.

**Figure 5:**
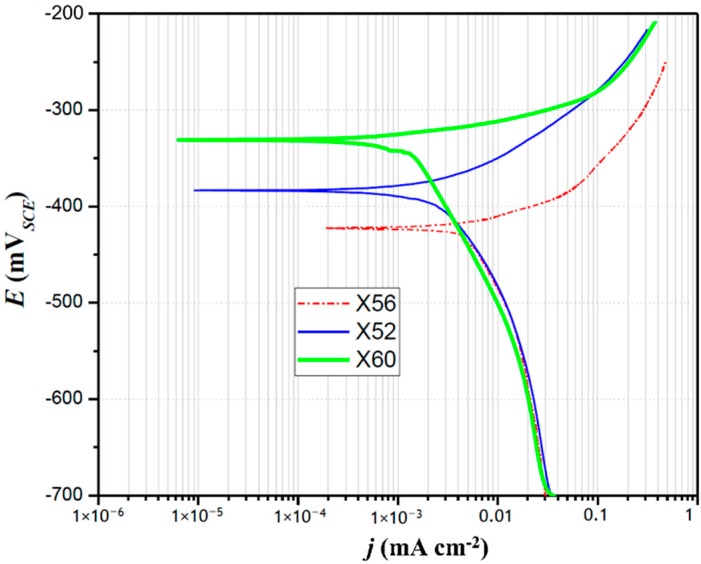
Potentiodynamic polarization curves of the three studied steel surfaces recorded at room temperature in the solution S** at a scan rate of 0.16 mV s^−1^.

Figure 5 shows the comparison of the potentiodynamic polarization curves recorded onto the three steels in the solution, S**. It can be observed in Figure 5 that the three investigated steels exhibited markedly different corrosion behavior as a consequence of their different textures and microstructures. The obtained *j*-*E* plots presented a characteristic Tafel behavior. The type of steel tested did not have an effect on the cathodic response, but did on the anodic branch. The X52 and X60 steels shifted towards potentials more noble, with respect to the X56 steel.

The corrosion potential, *E*_corr_, and current density, *j*_corr_, were determined using the extrapolation of the anodic and cathodic slopes, *b_a_* and *b_c_*; the resulting values are shown in Table 4. From a thermodynamic point of view, the more negative values of *E*_corr_ (X52 and X60 compared to X56) may be associated with greater corrosion tendency [6]. Therefore, as per the results of Figure 5 and Table 4, the corrosion tendency of the studied steels would increase in the following order: X60 < X52 < X56. This was in agreement with the CRI predictions made from the crystallographic texture. However, since the studied steels had different microstructural features and textures, the *E*_corr_ alone was insufficient to predict their corrosion behavior. As a result, a kinetic parameter, for instance *j*_corr_, was necessary. According to this parameter, the results of Table 4 clearly indicated that the X56 steel had a higher *j*_corr_ value than those of the X52 and X60 steels, confirming the following sequence of the CRI: X56 < X52 < X60. This follows the exact order of corrosion resistance or average CRI as had been previously predicted by the X-ray texture measurements, using Equation (12).

#### 4.3.2. Analysis of EIS Measurements

**Figure 6:**
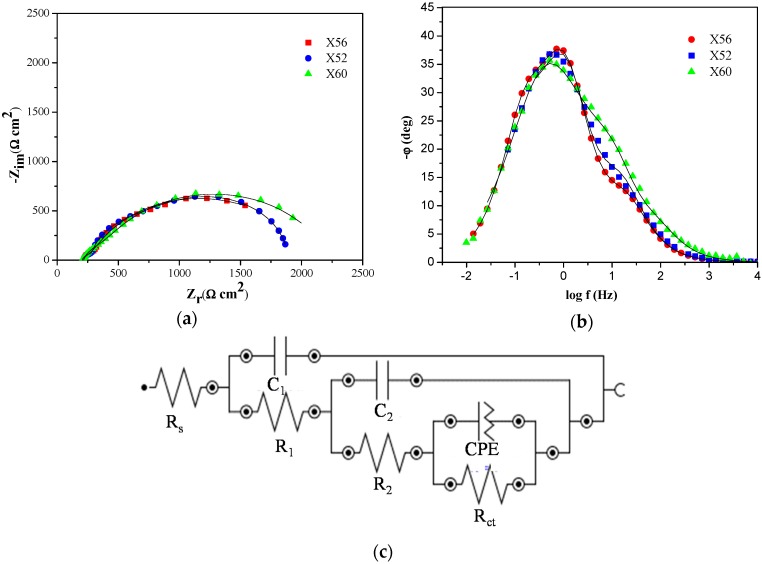
Electrochemical impedance spectroscopy (EIS) spectra of the studied steels in S** solution, (**a**) Nyquist complex diagram; (**b**) Bode phase angle diagram; and (**c**) equivalent circuit used to fit the EIS data.

The EIS data obtained in the S** solution for the three steels (X56, X52, and X60) are presented in Figure 6a,b in the form of Nyquist and Bode plots, respectively. To assist in the evaluation of the experimental results, an equivalent circuit model was developed, as shown in Figure 6c. The model consists of the solution resistance (*R*_s_) in series with the parallel combination of a resistance (*R*_1_) and capacitance (*C*_1_) associated to the film of the corrosion products; in series with a resistance (*R*_2_) and capacitance (C_2_) associated to the film pores that allow the charge transfer; and in series with the charge transfer resistance (*R*_ct_) and the constant phase element (*CPE*). The impedance of CPE, *Z*_CPE_, as a substitute of the capacitance of the ideal film due to its surface roughness and no uniformity in thickness, is given by [34]:

(13) $$Z_{\mathrm{CPE}}=Y_{0}^{-1}{\left(j\omega \right)}^{-n},$$

where *Y*_0_ is the admittance magnitude of the CPE; ω is the angular frequency (s^−1^), j is the imaginary unit (*j*^2^ = −1), and n is the *CPE* power (0 < *n* ≤ 1); depending on the values of *n* the CPE can reduce a capacitor (*n* = 1), a resistor (*n* = 0), an inductor (*n* = −1), or a Warburg element (*n* = 0.5).

Figure 6a reveals that the Nyquist diagrams approximated a single semi-circle, which could be associated with the charge transfer controlled process during the anodic dissolution of the steels. Meanwhile, the Bode plots in Figure 6b present a capacitive response with the formation of three phase maxima at high, intermediate, and low frequencies, which could be attributed to the properties of the formed corrosion films and their feature modifications. The presence of these capacitive loops indicates that several processes could take place due to the instability of the protective layer of corrosion products on the surface of the steel, which becomes weak and tends to dissolve. This allows the passage of ions from the solution and consequently generates a continuous degradation of the material.

The equivalent circuit model (Figure 6c) was used to fit the experimental data, using EIS analyzer software, to determine the kinetic parameters and assess the resistance of the material, which are shown in Table 5. *R*_ct_, for instance, was of interest. The radius of the semi-circles in Figure 6a and the *R*_ct_ values in Table 5 increased in the order X56 < X52 < X60, which corroborated the predictions of the CRI made from crystallographic texture and those obtained from the potentiodynamic polarization curves for the investigated steels.

It is important to note that the grain size could have been a factor that affected the corrosion behavior of the material, since X56 with the smallest grain size can be more corroded. However, Taleb et al. [28] verified that at a long time exposure, the effect of the grain size was negligible and, in most cases, the mechanism of pitting corrosion depends strongly on the crystallographic orientation.

In general, the good agreement between the average CRI predictions made from the crystallographic texture and the experimental results estimated from the potentiodynamic polarization curves verified the validity of the proposed and generalized model, and the strong effect that the crystallographic texture has on the corrosion behavior of the material. This influence can be explained due to the intimate and physical link between the surface roughness and crystallographic texture of the material. Additionally, it comes to support and extend the earlier idea proposed by Shahryari et al. [23] on estimating the corrosion resistance index in API 5L steels. Furthermore, the results open a new possibility to apply the proposed model as novel criterion for the selection and design of metallic structures, as well as an effective tool for the pipeline integrity strategy.

## 5. Conclusions

Corrosion is a complex phenomenon. One parameter that is important, particularly for the anodic dissolution of metal, is the crystallographic texture of the material. A novel model was proposed and validated for the estimation of the average corrosion resistance index from the crystallographic texture in API 5L steels, with different microstructures and textures. This model was based on the symmetric spherical harmonics, considering cubic (crystal)–orthorhombic (sample) symmetry with a large number of series of constants, and capable of reproducing the anisotropic corrosion behavior of the material. For all of the studied steels, strong correlations were observed between the predictions of the average corrosion resistance index, made from the crystallographic texture measurements, and the experimental results, from the potentiodynamic polarization and electrochemical impedance spectroscopy techniques. It was established that a material with a higher value of average corrosion resistance index has a lower corrosion current. The proposed and validated model provides a novel criterion for material selection. Furthermore, it is possible to apply it as an effective tool for the pipeline integrity strategy.

## Figures and Tables

**Table 1:** Ion concentrations for the solution used in this work.

Solution	Cl^−^ (ppm)	SO_4_^2−^ (ppm)	pH
S**	290	16.8	4.35 ^1^

^1^ pH was adjusted using HNO_3_ solution.

**Table 2:** Microstructural parameters of the investigated steels.

Steels	Average Grain Size (µm)	Ferrite Content (%)
X56	13.88 ± 0.012	75.45 ± 0.05
X52	18.22 ± 0.014	64.38 ± 0.03
X60	23.89 ± 0.015	65.42 ± 0.03

**Table 3:** Volume fraction of the texture fibers (in %) of the studied steels.

Materials	*V*_{001}_ND	*V*_{112}_ND	*V*_{111}_ND	*V*_{110}_ND
X56	8.32	22.29	12.34	3.93
X52	4.57	15.14	6.51	9.36
X60	5.37	16.68	7.44	9.19

**Table 4:** Corrosion parameters determined from the potentiodynamic polarization curves of the studied steels.

Materials	$\bar{E}\left(h\right)$	*E*_corr_ (mV_SCE_)	*j*_corr_ (µA cm^−2^)	*b_c_* (mV dec^−1^)	*b_a_* (mV dec^−1^)
X56	6.35	−460 ± 9	3.72 ± 0.05	284 ± 8	21 ± 0.5
X52	6.72	−385 ± 7	1.37 ± 0.03	284 ± 7	35 ± 0.6
X60	7.01	−338 ± 6	0.51 ± 0.01	294 ± 6	23 ± 0.5

**Table 5:** Parameter values obtained from the fitting of the EIS data with the equivalent circuit shown in Figure 6c for the studied steels.

Materials	*R*_s_ (Ω cm^2^)	*C*_1_ (µF cm^−2^)	*R*_1_ (Ω cm^2^)	*C*_2_ (µF cm^−2^)	*R*_2_ (Ω cm^2^)	*CPE* (µF cm^−2^)	*n*	*R*_ct_ (Ω cm^2^)
**X56**	217.4	3.09 × 10^−5^	122.2	1.02 × 10^−4^	818.2	1.58 × 10^−3^	1	729.5
**X52**	218.8	2.53 × 10^−5^	111.1	4.82 × 10^−5^	116.5	1.36 × 10^−4^	0.6730	1654.1
**X60**	206.9	1.32 × 10^−5^	71.2	2.36 × 10^−5^	201.7	1.76 × 10^−5^	0.7489	1758.1
